# Current Controversies in Lupus Anticoagulant Detection

**DOI:** 10.3390/antib5040022

**Published:** 2016-12-02

**Authors:** Gary W. Moore

**Affiliations:** Diagnostic Haemostasis & Thrombosis Laboratories, Department of Haemostasis and Thrombosis, Viapath Analytics, Guy’s & St. Thomas’ NHS Foundation Hospitals Trust, 4th floor North Wing, St. Thomas’ Hospital, Westminster Bridge Road, London SE1 7EH, UK; gary.moore@viapath.co.uk; Tel.: +44-020-7188-0814; Fax: +44-020-7188-2726

**Keywords:** activated partial thromboplastin time, antiphospholipid antibodies, antiphospholipid syndrome, dilute prothrombin time, dilute Russell’s viper venom time, lupus anticoagulant, mixing tests, Taipan snake venom time

## Abstract

Antiphospholipid syndrome is an autoimmune, acquired thrombophilia diagnosed when vascular thrombosis or pregnancy morbidity are accompanied by persistent antiphospholipid antibodies. Lupus anticoagulants (LA) are one of the criteria antibodies but calibration plasmas are unavailable and they are detected by inference based on antibody behaviour in a medley of coagulation-based assays. Elevated screening tests suggest the presence of a LA, which is confirmed with mixing tests to evidence inhibition and confirmatory tests to demonstrate phospholipid-dependence. At least two screening tests of different principle must be used to account for antibody heterogeneity and controversy exists on whether assays, in addition to dilute Russell’s viper venom time and activated partial thromboplastin time, should be employed. A variety of approaches to raw data manipulation and interpretation attract debate, as does inclusion or exclusion of mixing studies in circumstances where the presence of a LA is already evident from other results. Therapeutic anticoagulation compromises coagulation-based assays but careful data interpretation and use of alternative reagents can detect or exclude LA in specific circumstances, and this aspect of LA detection continues to evolve. This review focuses on the main areas of debate in LA detection.

## 1. Introduction

Antiphospholipid syndrome (APS) is diagnosed when laboratory assays demonstrate the presence of persistent antiphospholipid antibodies (aPL) in patients presenting with thrombosis or pregnancy morbidity [1]. Crucially, thrombosis and pregnancy morbidity are by no means specific to APS and diagnosis is highly reliant on accurate and timely detection of aPL. Solid phase assays are employed to detect two of the criteria antibodies, anticardiolipin antibodies (aCL) and anti-β2-glycoprotein I antibodies (aβ2GPI), whilst lupus anticoagulants (LA) are detected in coagulation assays.

Standardisation difficulties for aPL assays persist, arising from issues such as antibody heterogeneity, reagent variability and differing interpretation strategies, and so generation of gold standard assays and reference plasmas remains elusive [2,3]. Whilst aCL and aβ2GPI assays can be calibrated to generate quantitative results, the presence of LA is inferred based on antibody behaviour in a medley of phospholipid-dependent coagulation assays [4,5,6].

No single type of coagulation test is sensitive to all LA and two test systems of differing analytical principles should be employed to maximise detection rates [4,5,6]. Classically, the medley for each test type comprises:

(a) a screening test that employs a low phospholipid concentration to accentuate the effect of LA by increasing competition with activated coagulation factors for limited phospholipid-binding sites
(b) performance of the screening test on a 1:1 mixture of index and normal pooled plasma (NPP) to evidence inhibition
(c) confirmatory tests that recapitulate the screening test but with concentrated phospholipid to partially or wholly swamp/overwhelm any LA, thereby demonstrating phospholipid dependence

A patient with a LA and no other causes of elevated clotting times present would be expected to generate elevated clotting times in the screening test and mixing test, and a significantly shorter clotting time with the confirmatory test that typically, but not always, returns into the reference range. As long as the composite for one of the test systems is consistent with the presence of a LA, you have found what you are looking for even if the other has given a normal screening test result. One of the problems with employing global coagulation assays to infer the presence of LA is that standard interpretation criteria necessarily assume that all else about the patient’s coagulation status is normal, so each test has significant potential for compromised specificity, particularly in situations of therapeutic anticoagulation. This adds a further layer of complexity to LA identification and several guidelines with broad but not complete agreement are available to guide best practices [4,5,6,7]. The main interferences for each LA assay type are shown in Table 1.

## 2. Which Assays Should Be Used?

Numerous assay types for LA detection have been proposed and used over the years and earlier guidelines more or less gave practitioners free reign over which, and how many, to use, albeit with acknowledgement that the pairing of dilute Russell’s viper venom time (dRVVT) and activated partial thromboplastin time (APTT) can achieve good detection rates [1,8,9,10]. However, the most recent guideline from the International Society on Thrombosis and Haemostasis (ISTH), published in late 2009, suggests that that the risk of false-positive results is increased to unacceptable levels if more than two screening tests are performed and restricts assay choice to only dRVVT, for its specificity to clinically significant antibodies [11], and APTT with low phospholipid concentration because of its sensitivity [4]. This recommendation has its basis in the considerable body of evidence indicating that the dRVVT and APTT pairing is diagnostically efficacious, and additionally, it serves to nurture common diagnostic practices. An important caveat here is that not all reagents from different manufacturers for the same test perform identically, particularly in the case of APTT [7,11,12,13,14], and reagents for LA detection must be chosen carefully. Many APTT reagents intended for routine coagulation screening to primarily detect coagulation factor deficiencies and monitor heparin therapy do not contain dilute phospholipid and/or a suitable phospholipid composition, compromising their responsiveness to LAs. Available routine APTT reagents span a continuum of low, intermediate and (relatively) high LA sensitivity [14], whilst other APTTs are specifically formulated only for use in LA detection [15,16]. A valuable recommendation is made in the guideline from the Clinical and Laboratory Standards Institute (CLSI) from 2014 to employ a LA-insensitive APTT in routine coagulation screening and a separate, LA-sensitive reagent when specifically investigating for LA [6,17]. This permits interpretation of LA assays themselves unencumbered by the possibility of many interfering factors if the routine APTT is normal [6,7]. However, considerations of resource availability, cost and convenience lead many diagnostic facilities to employ one APTT reagent for both routine and LA testing, which can adversely affect diagnostic outcomes [14]. It has been suggested that ellagic acid-activated APTTs are less sensitive to LA and that only silica-activated reagents should be used [4]. This is based on reports comparing routine APTT reagents employing different activators [13] yet ellagic acid-activated APTTs that are LA-sensitive have been described [18] and the low sensitivity in the other reagents is merely coincidental to their phospholipid composition [18,19]. Utility of dRVVT in LA detection is indisputable, to such an extent that some laboratories erroneously employ it in isolation for LA detection [20,21,22]. Aside from recognition that not all LA will manifest in dRVVT, hence recommendations in all guidelines to employ at least two different tests [4,5,6,7,19], between-reagent variation exists for dRVVT reagents too [23,24,25,26], albeit to a lesser extent than APTT.

The issue of whether extending LA detection repertoires to include additional tests inevitably increases false-positivity rates is contentious [5,6,7,19]. Certainly, there will always be natural statistical outliers for any test regardless of the statistical model applied for cut-off generation, so in that sense, the more screening tests you employ the risk of false-positivity does indeed increase. However, any elevated screening test will subsequently receive mix and confirm tests, so weak LA that might be missed due to the adoption of a higher cut-off will not go undetected, and any genuine outliers will generate concordant confirm results and negative mix results. It is the composite that secures diagnosis, not an isolated screening test result, and it is for this reasoning that guidelines from the CLSI [6] and the latest from British Committee for Standards in Haematology (BCSH) [5] do not exclude performance of tests additional to dRVVT and APTT.

An important consideration regarding other tests is that assays such as dilute prothrombin time (dPT), activated seven lupus anticoagulant assay (ASLA), textarin time and Taipan snake venom time (TSVT) have been shown to detect small numbers of LA that do not manifest in dRVVT or APTT, or at least the versions of those two assays employed in a given study, as well as antibodies that do manifest in dRVVT or APTT [7,19,27,28,29,30,31,32]. Epitope specificity variation, even for antibodies to domain I of β2glycoprotein I [3,33], means that the dRVVT and APTT combination alone cannot deliver diagnostic certainty. Alternative assays are commonly evaluated against one or both of those assays, creating a selection bias, and are thus disadvantaged in the context of perceived utility because they may be sensitive to different antibody subpopulations. Antibodies unreactive in dRVVT and APTT can be clinically significant [27,28,29,30,34,35,36,37], yet it is probably impractical and unnecessary for all laboratories to adopt extended LA-assay repertoires. Nonetheless, there is a case for use of additional assays in select patients or circumstances [5,6,7]. It could be reasonably argued that this additional testing should be the remit of reference laboratories, particularly for less familiar assays, although recent reports have suggested that addition of the relatively straightforward dPT to a dRVVT and APTT pairing can improve detection rates [7,19,28,37]. Concern has been expressed about lack of standardisation with some of the alternative assays [4], which is not unwarranted for assays such as kaolin clotting time [6,7,19], but not insurmountable for assays such as dPT and TSVT where available reagents coupled with suitable expertise and experience can render them clinically valuable [28,29,32,34,37].

## 3. Raw Data Manipulation

The end-point for all LA assays is of course a clotting time in seconds. We are all familiar with reporting and interpreting routine prothrombin times and APTTs in seconds and some practitioners prefer to do so with LA assays [22,25,38], although all guidelines recommend conversion of screen, mix and confirm results to normalised ratios [4,5,6,7]. This practice can reduce intra-assay, inter-assay and between-laboratory variation by minimising operator and/or analyser variability, accounting for reagent quality and stability issues, and NPP clotting time variation [6,7,39,40]. The controversy concerns whether the NPP clotting time or reference interval (RI) mean clotting time should comprise the denominator. The advantage of the former is that NPP is analysed alongside patient samples, thereby accounting for innate between-run analytical variation by reflecting operator/reagent/analyser performance in real time. The disadvantage is that not all NPP generate the same clotting times with different reagents for the same test type, which can systematically bias calculated ratios towards false-positive or false-negative results if the NPP value is towards an extreme of the RI and distant from the RI mean [6,7,19,40,41]. Adopting the RI mean circumvents this potentially serious problem, but requires generation of a robust RI, and NPP samples should continue to be run alongside test samples to reflect real time assay performance and identify sudden analytical difficulties [7,14].

Another gain from normalisation is that clotting times for confirmatory tests are often shorter than those for screening tests, even in NPP [13,41], which risks interpreting a screen and confirm discordance as a LA when it is merely a function of reagent properties. Normalising the clotting times of donor plasmas in RI generation virtually abolishes these discrepancies such that screen and confirm RIs are almost identical [13,41] and thus permitting direct comparison of screen and confirm results in patient samples.

## 4. Generation of Reference Intervals/Cut-Offs

The plethora of available reagents and analysers means that common RIs and cut-offs cannot be applied to any given test and that locally derived reference ranges are necessary [4,5,6,7,23,39,40,41,42]. Historically, RIs for LA assays have been derived parametrically from the RI mean ±2 standard deviations (SD) since normal donor population data for clotting tests are commonly Gaussian, or can be made so by data transformation [5,6,7,13,43]. The RI upper limit operates as the cut-off for determining screening test positivity and initiating mixing and confirmatory tests, whilst the RI mean clotting time can be employed to generate normalised ratios.

Whilst common practice for these and many other assays, the consequent upper limit cut-off at the 97.5th percentile results in 2.5% of observations being above that cut-off and representing false-positive screening tests. To reduce this possibility, the current ISTH guideline recommends application of the 99th percentile as the cut-off, which equates to the RI mean +2.3 SD for normally distributed data. This can reduce frequency of false positive results and thereby increase specificity, yet it is a statistical inevitability that this will reduce sensitivity [5,6,7]. The recommendation has proven controversial, partly because composite testing will identify any false-positive screening results and the slight reduction in sensitivity is therefore avoidable, but also because it is indicated that a 99th percentile value can be derived from a minimum of 40 donors when a minimum of 120 has been previously recommended [44]. Non-normally distributed data require considerably more. Sourcing that many normal donors is problematic and impractical for most diagnostic facilities and advice in the BCSH and CLSI guidelines maintains that generating a RI from its mean ±2 SD remains a valid and achievable proposition, and can be done with as few as 20 donors through validation exercises of previous cut-offs. The theoretical loss of sensitivity when adopting 99th percentile cut-offs has been substantiated in recent studies [45,46,47] and some workers continue to adopt 97.5th percentile, and even 95th percentile [41,46,47,48].

## 5. Confirmatory Tests

Despite recommendations in earlier as well as current guidelines, many laboratories have limited their APTT testing to screen and mix tests only, further restricting specificity of that assay system [22,49]. Indeed, the design and interpretation of the previously widely used kaolin clotting time, an APTT modification, is predicated on mixing test results [1,10]. This was partly due to poor availability of paired APTT confirm reagents [6]. Reports that pairing a known LA-sensitive APTT with an known insensitive reagent where practitioners ostensibly just need to perform two routine APTTs [14,17,48] have led to a greater but not complete uptake of APTT confirm assays [20,21,22,49]. Another approach is to screen with a LA-sensitive reagent, and if elevated, proceed to testing with the Staclot^®^ LA assay. This involves performing APTTs on equal volume mixtures of index plasma and NPP with a separate, highly LA-sensitive reagent in the absence or presence of hexagonal phase phosphatidylethanolamine, the latter being the confirmatory test [6,15].

Antibody heterogeneity makes it crucial that confirmatory tests are based on whichever screening test is elevated [4,5,6,7,8,9,10]. Finding an elevated APTT screening test but attempting to discern phospholipid dependence by performing dRVVT screen and confirm assays will result in failure to accurately identify a LA that is dRVVT-unreactive.

Confirmation of phospholipid dependence is achieved by mathematically establishing that any difference between screen and confirm values is significant and due to more than analytical variance. Although the BCSH guidelines from 1991, 2000 and 2012 recommend the percent correction of screen ratio by confirm ratio for dRVVT, where >10% correction is considered significant, a variety of other calculations to assess for a significant difference have been proposed and used [9,50]. The issue was not directly addressed by ISTH guidelines until the 2009 update, where percent correction is also the recommendation, accompanied by a recommendation to assess the cut-off locally [4,8,10]. However, regulatory issues in some countries restrict practice to directions given by manufacturers in their package inserts and a common instruction is to calculate a ratio from dividing the screen result by the confirm result and assess against a cut-off [6,25]. Clearly, a high ratio is indicative of phospholipid dependence, yet this is often derived from raw clotting times without prior screen and confirm normalisation, which risks misclassification due to reagent-induced differences, and reduction in inter-method and inter-laboratory agreement [6,13,25]. In recognition, all current guidelines indicate that laboratories adopting this approach should first normalise screen and confirm results [4,5,6,7]. Some APTT-based assays, such as Staclot^®^ LA, are assessed with deltas [6,15].

An interesting consequence of the availability of paired screen and confirm reagents is the adoption of so-called integrated testing where the reagent pair are assayed in parallel with each patient [4,7,11,25,48]. This permits immediate assessment for phospholipid dependence and circumvents the traditional algorithm of performing a mixing test in response to an elevated screening test and initiation of a confirmatory test if the mix is also elevated. One potential benefit is improved detection of weaker LA where the prolongation of a patient’s basal screening test clotting time is insufficient to exceed the cut-off yet the screen and confirm discordance elevates the ratio, or indicates high percentage correction, and reveals the antibody [51,52,53]. The immediate availability of demonstration of phospholipid dependence has led some to question the role of mixing tests in the LA detection armoury [6,7,49,51,53,54,55,56,57], to which we will now turn our attention.

## 6. Mixing Tests

Lupus anticoagulants are, by definition, in vitro inhibitors, so it is entirely logical to investigate an elevated screening test with a mixing test in the first instance. If an elevated screening test does not prove to be due to an inhibitor it wastes time and resources to perform the confirmatory test. Or does it? Whilst that was accepted wisdom and standard practice for some time it is now widely accepted, and acknowledged in guidelines, that mixing tests introduce a dilution factor that can generate false-negative results such that adopting the traditional screen-mix-confirm algorithm may reduce detection rates [4,5,6,7,51,57,58,59,60]. This has fuelled acceptance and the adoption of the integrated testing model as a faster and more cost-effective LA detection strategy, yet it does not represent the full picture and returns us to the issue of specificity.

As stated in the BCSH guideline [5], a patient with clear screen and confirm discordance in undiluted plasma and no other causes of elevated clotting times but with a normal mixing test can be reasonably considered to have a LA, the mixing test result being merely due to a limitation of test design. Evidence for absence of a co-existing abnormality largely comes from the coagulation screen employing a LA-insensitive routine APTT, if normal, but also from the confirmatory test whose partner screening test was elevated, in that a normal confirmatory test is additional evidence that interfering factors specific to that test system are not present [6,19,54,61]. However, if significant screen and confirm discordance is apparent but the confirm result is elevated, consideration must be given to the possibility of a co-existing abnormality such as a factor deficiency or undisclosed therapeutic anticoagulation that could compromise LA detection [6,7,26,54,61,62]. Alternatively, some LA possess a degree of resistance to the swamping effect of confirm reagents and the confirm result is elevated for this reason alone [4,7,54]. In such circumstances, mixing tests can be invaluable by confirming the presence of inhibition or correcting for some non-LA abnormalities. Specificity can be further increased if a confirm mixing test is also performed as it aids discrimination between potent LA, LA + co-existing abnormality or non-LA abnormality [1,7,54,63,64]. Non-LA causes of elevated clotting times usually generate concordant screen and confirm ratios and integrated testing generates a normal ratio or low percent correction, thereby excluding a LA. However, exceptionally potent LA may also generate such results when tested with undiluted plasma and the dilution factor in mixing tests can be used to diagnostic advantage by dampening the effect of the antibody and reveal screen and confirm discordance [19,54,55,64]. The important message here is that some LA can be reliably detected without mixing tests and the decision point for progressing to mixing tests is whether or not the confirm result from undiluted plasma is elevated [6,7,19,54,61]. This has led the CLSI to recommend a re-prioritisation of testing order to screen-confirm-mix, the mixing tests only being performed when other tests are not clear cut and the decision is made on a case-by-case basis.

Assays with higher specificity tend to require less mixing tests to clearly demonstrate LA [56,65]. Although considered rare, it is inevitable that the LA cofactor effect cannot be detected when mixing tests are omitted [7,55]. The LA cofactor effect is the paradoxical further prolongation of the patient’s clotting time upon mixing with NPP [6,10,55]. The phenomenon is thought to be due a patient’s plasma being deficient in an as yet undefined cofactor that is essential for LA to exert their in vitro anticoagulant effect. The NPP normalises the cofactor level, thereby permitting greater expression of the LA-induced inhibition. The cofactor has been proposed but not proven to be prothrombin or β2-glycoprotein I [6].

Accepting that mixing tests maintain a valuable role in LA detection but are compromised by the dilution effect necessitates exploration of strategies to maximise detection rates. Adopting dilutions that favour an excess of index plasma has been proposed but does not guarantee correction of severe or multiple factor deficiencies [6,10], and although potentially useful in detecting weaker antibodies [66], it is resource expensive and practically cumbersome to perform multiple mixing test dilutions. Instead, efforts have been centred on alternative approaches to interpretation of 1:1 mixing tests. It is common to interpret mixing tests for standard coagulation tests against the RI for undiluted plasma [67] but mixing test-specific RIs for LA assays have been shown to be narrower than for undiluted plasma due to clotting times of normal samples at the extremes being compensated upon mixing with NPP [6,7,11,13,60]. The narrower RI and thus lower cut-off increases sensitivity for inhibition [6,13,38,60,68] and is now recommended for interpreting LA mixing tests [4,6,7]. The index of circulating anticoagulant (ICA) calculation [46,69] is an alternative recommendation [4,6], although recent studies have reported that a mixing test-specific cut-off is more sensitive than ICA in detection of inhibition [38,68].

## 7. Testing Anticoagulated Patients

All therapeutic anticoagulants have potential to compromise LA testing with some or most available assays and laboratory investigation for LA is best postponed until after discontinuation of anticoagulant treatment [4,5,6]. Despite this, it is common for samples from anticoagulated patients to be submitted for diagnostic LA testing [7,70]. It is therefore incumbent on diagnostic laboratories to recognise and maximise situations where LA can nonetheless be detected, yet be honest with themselves and their service users when they cannot. This would normally take the form of performing the assays on each patient and interpreting in light of assay design and limitations, degree of anticoagulation, reagent properties and whether strategies such as mixing studies employing both screen and confirm assays, and heparin neutralisers, have accounted for anticoagulation interference.

### 7.1. Vitamin K Antagonists

An almost perennial controversy is whether LA assays performed on undiluted plasma from patients receiving vitamin K antagonist (VKA) anticoagulation are reliable, particularly dRVVT [57,71,72,73,74]. Some contend that the multiple acquired factor deficiency of VKA therapy does indeed compromise LA detection [71,73], whilst others maintain that any screen and confirm discordance is reliable despite any confirm test elevation [51,72]. Isert et al. proposed an elevation of the dRVVT normalised screen/confirm ratio cut-off from 1.3 to 1.7 when testing plasma from VKA anticoagulated patients (International Normalised Ratio (INR) 2.0–3.0), which improved accuracy in known APS patients but risked a mild reduction in sensitivity [74]. In view of the controversy, others choose to disregard results from undiluted plasma and instead rely on mixing tests where an elevated screen evidences inhibition, and a reduced and often normal confirm evidences that the mix corrects the VKA effect and the reagent corrects the LA [1,5,6,63]. A positive result is diagnostic but negative mixing tests are inconclusive due to the dilution effect [1,5,6].

An additional tool for detecting LA in VKA anticoagulated patients is use of assays based on ‘VKA-insensitive’ snake venom prothrombin activators [31,34]. Textarin and Taipan venoms are phospholipid-dependent, so diluting the phospholipid makes the assays LA-responsive, and employing prothrombin activators aids specificity. In place of concentrated phospholipid confirmatory tests, both venoms are commonly paired with ecarin venom, which contains a phospholipid-independent, ‘VKA-insensitive’ prothrombin activator [31,32,34,63,75]. Combining TSVT and ecarin time (ET) with dRVVT and APTT mixing tests increases detection rates, partly because antibodies ‘lost’ by dilution can still manifest in TSVT/ET [5,6,63], and also because TSVT will detect a small proportion of LA unreactive with dRVVT and APTT [32,34,63,76].

It has been suggested that that there are no standardised commercial assays employing these venoms [4], and whilst true for Textarin, paired TSVT and ET reagents have been available for some time from at least one manufacturer [7,32,34]. The ISTH scientific sub-committee chose not to recommend these assays for use in VKA anticoagulated patients because it was considered they required further critical evaluation in that setting [4]. Both BCSH and CLSI guidelines do however suggest they can be used in this patient population, possibly because authors on those committees had more direct personal experience of them [5,6,7,34,63,76].

An important consideration for patients with LA who are treated with VKA anticoagulation is that they are monitored with a phospholipid dependent test, the prothrombin time (PT), to generate the INR. Fortunately, the high phospholipid concentration in thromboplastin reagents means that >95% of patients with APS have a normal PT in the absence of other coagulopathies [5]. For patients whose LA does prolong the locally employed PT prior to anticoagulation, thereby risking overestimation of anticoagulation, monitoring with an alternative, LA-insensitive thromboplastin shown to generate a normal baseline for that patient is usually achievable. More rarely, amidolytic factor X assays can be employed [1,5]. Reagents comprising recombinant tissue factor and purified phospholipids tend to be slightly more prone to LA interference. Additionally, point-of-care devices for INR generation can also be affected by LA and baseline PT on the device should be performed prior to commencement of anticoagulation [5].

### 7.2. Heparins

Interference by unfractionated heparin (UFH) is inevitable in LA assays and attempting to detect the antibodies in this situation is largely discouraged [4,5,6,7]. However, most commercial dRVVT reagents contain heparin neutralisers that are effective up to a specified UFH level, commonly between 0.8 and 1.0 U/mL. The important question for practitioners who interpret these results is how they know the neutraliser has quenched the UFH without simultaneously assaying UFH levels. Once again, the confirmatory test comes to our rescue. An elevated screen can be attributed to a LA if the confirm ratio is normal since its phospholipid has corrected the LA and the neutraliser has dealt with the UFH [6]. Assays employing confirmatory tests derived from platelet material should not be used as the platelet factor 4 will neutralise UFH and can generate false-positive screen and confirm discordance [5,6,77].

Low molecular weight heparins (LMWH) are generally considered to have little or no effect on standard prothrombin time and APTT assays yet dose, LMWH type and reagent variability can generate elevated clotting times [78,79]. Although interference of LMWH in LA assays is less frequent than with some other anticoagulants it does occur and the presence of LMWH must be taken into account when interpreting LA assays, even in reagents with heparin neutralisers [6,62,80].

### 7.3. Direct Oral Anticoagulants

The direct oral anticoagulants (DOAC) inevitably complicate LA testing and interpretation, and data continue to emerge concerning their effects on different LA assays [62,80,81,82,83,84]. Numerous studies have reported greater elevation of dRVVT screen values than confirm values with direct factor Xa (DFXa) inhibitors in non-LA patients or spiked NPP [26,62,81,83,84,85,86,87]. The screen and confirm discordance is sufficient to generate almost ubiquitous false-positivity at peak rivaroxaban concentrations, and to a lesser extent at trough levels [82]. Similar patterns are seen with edoxaban but to a lesser extent with apixaban [81,86]. Being direct inhibitors, mixing tests are also elevated, further increasing the risk of false-positive interpretations. As might be expected, APTT testing for LA is less often affected by DFXa inhibitors but caution is required when interpreting results as reagent variability exists [62,81,86,87].

These complications can be overcome with the TSVT/ET pairing since the direct prothrombin activation by both venoms bypasses the effects of DFXa inhibitors. Recent studies have reported successful LA detection with TSVT/ET in patients on rivaroxaban [75,84,88,89]. However, TSVT/ET alone will not detect all LA and additionally employing APTT-based assays that are insensitive to DFXa inhibitors [81] should help account for antibody heterogeneity. Newly developed dRVVT reagents that are less affected by warfarin and rivaroxaban, and thus exhibiting improved specificity, have recently become available and been evaluated [26,81,88]. Although both therapeutic anticoagulants still elevate the screen values, patients without LA generate concordant confirm values and the false-positive interpretations encountered with most other dRVVT reagents do not ensue. Another recent study reported similar findings with home-brew dRVVT reagents [84]. There does appear to be a slight loss of sensitivity with the commercial reagents, albeit no less sensitive than some other commercially available dRVVT reagents [25,26,88]. In view of this, a panel of DOAC-insensitive APTT, VKA and DOAC insensitive dRVVT and TSVT/ET could maximise detection rates in anticoagulated patients.

Being a direct thrombin inhibitor, dabigatran potentially interferes with all LA tests and false positive interpretations are common, including elevations of mixing tests [62,80,82,90,91,92]. Testing for LA is best postponed until dabigatran is withdrawn, although a recent study reported successful in vitro idarucizumab-induced reversal of dabigatran anticoagulation in dRVVT and other routine coagulation tests [92].

## 8. Can Lupus Anticoagulants Be Quantified?

The current detection of LA by inference in coagulation assays provides evidence of presence but not concentration. The ability to quantify LA could help stratify patients into risk groups but is complicated by the lack of a true plasma standard with assigned activity since the antibodies are heterogeneous [93]. This heterogeneity, coupled with between-reagent variability, can result in a given LA appearing to be a strong positive with one reagent but weak or even negative with another [12,17,20,22,50], making even semi-quantitative assessment of results as weak, moderate or strong almost meaningless.

An early attempt to quantify LA involved performing APTT and dRVVT screen and confirm assays on 1:1 mixtures of test plasma and NPP. The ratio between the two clotting times in each test was divided by the corresponding ratio for the NPP itself, generating a third ratio referred to as the Lupus Ratio [94]. The upper limit of a normal reference population was deemed to represent one LA Unit (LA-U) and dilutions of a single ‘strong’ LA-positive plasma used to construct calibration curves. Although exhibiting high sensitivity and specificity, use of a single plasma for calibration was a limitation and the Lupus Ratio alone was later proposed as a semi-quantitative procedure [95].

Perhaps unsurprisingly, other workers have explored spiking NPP with antibodies known to possess LA activity. Le Querrec et al. demonstrated that various clotting assays could be calibrated against a NPP spiked with monoclonal antibodies to the two most common antigenic targets of LA, β2-glycoprotein I and prothrombin [96]. However, there were large differences in responsiveness between the assays and reagents employed, and the two monoclonal antibodies would not necessarily account for the polyclonal nature of LA or other antigenic targets such as annexin A5 [97].

Tripodi et al. performed a feasibility study for an alternative approach involving assignment of a LA sensitivity index (LASI) to LA assay reagents directly analogous to the international sensitivity index used for INR determination [98]. The usual ratios generated from LA testing were converted into a new ‘universal’ scale called standardised LA-ratio (SLA-ratio). Reagent differences manifested when SLA-ratio was calculated from LASI calibration with NPP spiked with purified IgG from patients with ‘strong’ LA and aβ2GPI but were abrogated when calibration was against a set of plasmas from LA-positive patients.

Possibly the most promising approach has been to generate a ratio from the peak height and lag time thrombin generation parameters and calibrate against NPP spiked with monoclonal antibodies to β2-glycoprotein I and prothrombin to quantify LA activity in arbitrary units [93]. Although the above mentioned limitations with this calibration material remain, combining the quantified LA results with those of other assays, such as the solid-phase assay aβ2GPI titre and factor VII activity, permitted a layered strategy for thrombotic risk assessment, but which was not possible with the thrombin generation parameters alone.

Can lupus anticoagulants be quantified? Not quite, but we are getting ever closer, although generating that polyclonal plasma standard applicable to all patients and antigens remains a high hurdle.

## 9. Conclusions

The publication of recent guideline updates by the ISTH and BCSH, and the new CLSI guideline, have gone a long way towards harmonising diagnostic practices, despite incomplete agreement on certain issues [7,19,37,40,45,65,70]. Most laboratories will likely continue to employ only the dRVVT and APTT pairing, which will detect most LA, yet experienced proponents of other assays are unlikely to be deterred from using them as they have evidenced genuine clinical utility, at least in certain circumstances [28,34,37,75,84]. Although the mixing test debate will continue apace [7,13,49,51,54,55,56,57,59,61], many diagnostic departments have already made their decision whether to retain this member of the medley or not [45,49,56,57,61,63,68], despite advice that it is invaluable in some situations and probably unnecessary in others. Until the unlikely event that LA calibration plasmas become available, practitioners will continue to apply their preferred statistical model for cut-off generation with respect to the balance between sensitivity and specificity. Consensus has almost been reached on how to evidence phospholipid dependence, with most laboratories applying percent correction or normalised screen/confirm ratio. Similarly for mixing tests, although recent reports suggest mixing test cut-off is more sensitive than ICA in detecting inhibition in multiple assays [38,68,99] and additional studies may consolidate this view sufficient for a firm recommendation in future guidelines.

Detection of LA in anticoagulated patients is possible in many cases upon careful application of appropriate assays and interpretation strategies. It is conceivable that DOAC and VKA insensitive reagents, and/or addition of DOAC reversal agents, will greatly improve this situation in the near future.

Recognition of the importance of antibodies to domain I of β_2_-glycoprotein I in the pathophysiology of APS [33,100], and recent availability of solid phase assays to detect them [101], may have posed a threat to the continued use of coagulation assay medleys to detect clinically significant aPL [7]. However, antibody heterogeneity, proven clinical utility, and detection of clinically significant LA without β_2_-glycoprotein I specificity will keep coagulation-based LA assays in diagnostic repertoires for the foreseeable future.

## Figures and Tables

**Table 1:** Main interfering factors in lupus anticoagulant assays affecting specificity.

Assay Types	Assays	Interferences
Intrinsic pathway-based assays	LA-responsive routine APTT Dilute APTT KCT SCT	*Non-LA causes of screening test elevation* Deficiencies of factors II, V, VIII, IX, X, XI, XII, PK, HMWK Reduced fibrinogen Anticoagulation with VKA, UFH, (LMWH), DFXa, DTI Non-phospholipid-dependent inhibitors *Shortening of screening test* Elevated FVIII, FIX Elevated fibrinogen
Extrinsic pathway-based assays	dPT ASLA	*Non-LA causes of screening test elevation* Deficiencies of factors II, V, X; (dPT only: factors VII, VIII, IX) Reduced fibrinogen Anticoagulation with VKA, DFXa, DTI, UFH Non-phospholipid-dependent inhibitors
Common pathway-based assays FX activation	dRVVT VLVT	*Non-LA causes of screening test elevation* Deficiencies of factors II, V, X Reduced fibrinogen Anticoagulation with VKA, DFXa, DTI, (UFH, LMWH) Non-phospholipid-dependent inhibitors
Common pathway-based assays FII activation	TSVT Textarin time	*Non-LA causes of screening test elevation* Deficiency of factor II; (Textarin time only: factor V) Reduced fibrinogen Anticoagulation with UFH, LMWH, DTI Non-phospholipid-dependent inhibitors

APTT, activated partial thromboplastin time; ASLA, activated seven lupus anticoagulant assay; DFXa, direct activated factor X inhibitor; dPT, dilute prothrombin time; dRVVT, dilute Russell’s viper venom time; DTI, direct thrombin inhibitor; HMWK, high molecular weight kininogen; KCT, kaolin clotting time; LMWH, low molecular weight heparin; PK, prekallikrein; SCT, silica clotting time; TSVT, Taipan snake venom time; UFH, unfractionated heparin; VKA, vitamin K antagonist; VLVT, *Vipera lebetina* venom time.
